# Isoprostanes as biomarkers of oxidative stress across human diseases and skin pathologies

**DOI:** 10.3389/fmolb.2026.1819898

**Published:** 2026-08-04

**Authors:** Aleksandra Okuniewska, Narcyz Knap, Ewa Kulpińska, Michał Woźniak, Alicja Dębska-Ślizień, Beata Imko-Walczuk

**Affiliations:** 1 Department of Medical Chemistry, Medical University of Gdansk, Gdańsk, Poland; 2 Dermatology and Venereology Outpatient Clinic, Copernicus, Independent Public Healthcare Centre, Gdańsk, Poland; 3 Department of Nephrology Transplantology and Internal Diseases, Medical University of Gdańsk, Gdańsk, Poland

**Keywords:** biomarkers, cancer, human disease, isoprostanes, oxidative stress, skin cancer, skin pathologies, transplantation

## Abstract

Oxidative stress (OS), resulting from an imbalance between reactive oxygen species (ROS) production and antioxidative defenses, is connected with various diseases, including cancer. Isoprostanes (IsoPs), derived from peroxidation of polyunsaturated fatty acids (PUFAs), serve as reliable biomarkers of OS. This review collects knowledge about IsoPs as indicators of OS, focusing on their association with various diseases, among others different types of cancer, and also skin disorders. Roughly 3 decades ago IsoPs were characterized *in vivo*. It represented a significant milestone in the field of biomedical sciences, and specifically in biochemistry of lipid peroxidation and free radical research. Between 1992 and 2024, more than 5,800 articles on IsoPs were published. IsoPs, for example, 8-iso-prostaglandin F2α, are relatively stable indicators of OS. Elevated IsoPs levels are associated with various cancers, cardiovascular, metabolic, and neurodegenerative diseases. There are some articles about IsoPs and their correlation with diseases like diabetes, asthma and colorectal, breast, lung or nonmelanoma skin cancer. Despite their broad relevance, a gap exists in the literature regarding IsoPs and skin cancer, especially in a very specific group of patients like organ transplant recipients, who are at a much heightened risk of developing cancer due to immunosuppression. IsoPs are valuable biomarkers for OS-related diseases. However, further research is needed to explore their pathophysiological significance and potential clinical applicability with regard to skin cancer risk or diagnosis in transplant patients, aiming to improve disease prevention, monitoring and therapeutic approach.

## Introduction

Oxidative stress (OS) is connected with many pathologies (Tkaczyk and Vizek, 2007; Fam and Morrow, 2003; Sies, 1985). It occurs when balance between production of reactive oxygen species (ROS) and antioxidative defense is disturbed in favor of the former (Sies, 1985). Peroxidation of phospholipids in biological membranes often results in cellular damage. It is suggested that oxidative stress is connected with inflammation and affects cell growth. This phenomenon can lead to many pathological conditions, and among others to carcinogenesis. Nowadays, we know that there are certain low-molecular compounds which can be used for finding a correlation between oxidative stress and its role in the process of cancer induction and development. One of them is isoprostanes (IsoPs) (Fam and Morrow, 2003; Andries et al., 2021). They are prostaglandin-like molecules which are products of peroxidation of polyunsaturated fatty acids (PUFA). IsoPs are common in biological fluids like urine, blood, cerebrospinal fluid, or are present in expired breath gas. These days, researchers not only utilize IsoPs as a biomarker of health disorders associated with oxidative stress, but also keep looking for a possibility of their application to monitor changes observed in the organism which are caused by external factors, e.g., by xenobiotics like drugs or toxins. In the years 1992–2024 more than 5,800 articles about IsoPs were published.

## Aim

This article presents collected knowledge focusing on publications dealing with IsoPs as markers of oxidative stress and their association with certain diseases, especially cancer, with particular emphasis on skin cancers also developing in transplant patients.

## Methods

The screening procedure aimed at finding data about isoprostanes as biomarkers of OS in various diseases, including skin disorders was based both on literature research and authors’ own experience based on performed clinical trials. The PubMed database was searched using different variations of key words like „isoprostanes” and/or „biomarkers” and/or „oxidative stress” and/or „skin cancer” and/or „cancer” and/or „disease”. The literature resources were searched until the year 2024. Additional articles were identified through reference search of the articles obtained via PubMed.

## Isoprostanes

### Formation and biochemical mechanisms

Isoprostanes are products of mostly nonenzymatic PUFAs peroxidation induced by ROS, i.e., via biochemical pathways independent of the enzymatic activities of Cyclooxygenase-1, -2 or -3 (COX-1, COX-2, COX-3). Interestingly, isoprostanes like prostaglandins being actually their stereomeric analogs, may also show biological activities (Fam and Morrow, 2003; Knap et al., 2010; Comporti et al., 2008).

### Biological activities of isoprostanes

IsoPs act as vasoconstrictors (Knap et al., 2010; Renke et al., 2009), affecting arterial blood pressure (Renke et al., 2009; Renke et al., 2008) and kidney function (Renke et al., 2013; Renke et al., 2014), induce mitogenesis in vascular smooth muscle cells and modulate blood platelets (Fam and Morrow, 2003). They are commonly associated with all tissues and biological fluids. They are either esterified within cell membrane phospholipids or present as free compounds in tissues or body fluids.

Nowadays, the best known and studied IsoPs are derivatives of arachidonic acid which are prostaglandin isomers.

### Clinical relevance of isoprostanes

Their omnipresence in human biological fluids as well as relative chemical stability, make them one of the most reliable markers of oxidative stress and lipid peroxidation *in vivo*. Measuring the concentration of these substances has a prognostic value in diseases whose etiology is associated with OS, for example, certain types of cancer, and other diseases relating to the respiratory tract (Tkaczyk and Vizek, 2007), cardiovascular system (Renke et al., 2009), kidneys (Renke et al., 2008; Renke et al., 2010a), as well as pathologies observed in pregnancy like, e.g., preeclampsia (Zabul et al., 2015; Brien et al., 2017), metabolic disorders (Renke et al., 2010b; Mukhtar et al., 2016) and neurodegenerative diseases (Klarzynska et al., 2017; Trares et al., 2022).

**FIGURE 1:**
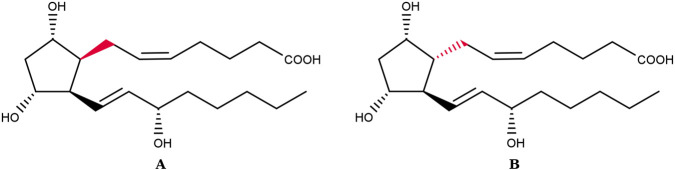
Stereochemical structure of 8-iso-prostaglandin F2α **(A)** compared with prostaglandin F2α **(B)**. Solid wedges represent hydrocarbon chains being both elevated above the averaged plane of the cyclopentane ring, and thus achieving a *cis* configuration rather than the *trans* configuration typical for prostaglandins which are geometric isomers with respect to isoprostanes (critical bonds making the difference are highlighted in red). Structures created and optimized with MarvinSketch 23.4.2, ChemAxon (Academic Research License).

One of the IsoPs is 8-iso-prostaglandin F2α (Figure 1) which is often considered the best marker of OS (Havet et al., 2018; Van’t Erve et al., 2016).

Because oxidative stress and lipid peroxidation contribute to inflammation, carcinogenesis and tissue injury, increasing attention has also been paid to natural antioxidant compounds and dietary factors potentially modulating redox homeostasis. Various natural products including curcumin, propolis, honeybee-derived compounds and plant-derived antioxidants have been investigated for their antioxidative and immunomodulatory properties in different pathological conditions, including inflammatory disorders and cancer (Tabrizi et al., 2019; Saad et al., 2026; Elumalai et al., 2022; Pasupuleti et al., 2017).

### Identification of isoprostanes

The first report on the possibility of nonenzymatic PUFA oxidation was presented in 1967, when Nugteren et al. observed formation of prostaglandin E1 (PGE1) as a result of autoxidation of dihomo-gamma-linolenic acid *in vitro* (Basu, 2004; Nugteren et al., 1967). A similar occurrence was reported later regarding arachidonic acid (AA). Derivative products of AA autoxidation had structures similar to PGs (Lawson et al., 1999). This observation gained much more importance in 1990, when Morrow et al. (Morrow et al., 1990) discovered formation of these derivatives as a result of nonenzymatic peroxidation *in vivo*. Nowadays, these compounds are known as F2-IsoPs (Morrow et al., 1990; Halliwell and Lee, 2010). For a long time researchers thought that nonenzymatic lipid peroxidation was the only possible source of F2-IsoPs in the biological system (Van’t Erve et al., 2016; Kadiiska et al., 2005a).

As of now, it is known that some IsoPs can also be produced in the enzymatic pathway of AA conversion dependent on prostaglandin endoperoxide-H synthase (PGHS) type I and II also referred to as COX-1 and COX-2. The results obtained by researchers from NIEHS (National Institute of Environmental Health Sciences) show that IsoPs as products of the enzymatic pathway can be a significant part of the total pool of these compounds. Percentage of enzymatic and nonenzymatic fraction of IsoPs depends on the type and intensity level of the factor inducing OS (van 't Erve et al., 2015). According to van ‘t Erve et al., the measure of OS *in vivo* should be actually evaluated as a ratio between 15-F2t-isoprostane (15-F2t-IsoP) and prostaglandin F2α (PGF2α).

### Isoprostanes as biomarkers in oxidative stress and disease

In 2017, van ‘t Erve et al. presented a powerful meta-analysis which explained correlations between oxidative stress and several diseases (van 't Erve et al., 2017). They chose 8-iso-PGF2α as a representative biomarker of oxidative damage and classified the level of that marker across 50 human health outcomes and exposures from 242 distinct publications. They claimed that certain pathologies like hypertension, asthma, metabolic syndrome and cigarette smoking are connected with a relatively small increase of F2-IsoPs level in comparison to other conditions such as kidney pathologies or respiratory tract disorders. However, there are very many reliable publications from real experts in the field, which claim otherwise observing dramatically higher levels of IsoPs in very many pathologies associated with oxidative stress, such as cancer, cardiovascular, metabolic and neurodegenerative diseases (Morrow et al., 1990; Kadiiska et al., 2005a; Morr et al., 1992; Bauer et al., 2014; Roberts and Morrow, 2000).

## Isoprostanes in metabolic and cardiovascular disorders

### Diabetes mellitus

Pękala-Wojciechowska et al. conducted a comparative study checking a concentration of 8-isoprostane in the exhaled breath condensate (EBC) as a marker of OS among patients with type 1 diabetes mellitus (Pękala-Wojciechowska A et al., 2018). They concluded that this kind of IsoP measurement was not a good diagnostic method for monitoring OS in this group of patients. Blood levels of 8-IsoP were significantly higher compared to the control group. However, the mean concentration of 8-isoprostane in EBC was lower in diabetic patients with type 1 diabetes with advanced complications than in patients with type 1 diabetes without advanced complications as well as in the control group. According to the authors’ conclusion, IsoPs in exhaled air are not a suitable biomarker that could be used to monitor type I diabetes (Pękala-Wojciechowska A et al., 2018). Interestingly, a cross-sectional analyses in a sample of premenopausal women proved significant relations between high dietary glycemic index and glycemic load with oxidative stress as assessed by urinary F2-IsoP concentrations (Anderson et al., 2018a). Such observations are of clinical importance as they link dietary abuse of simple carbohydrates with protein glycation, and consequent chronic oxidative stress potentially leading to the development of cardiovascular disease and type 2 diabetes. In another study, serum levels of 8-iso-PGF2α and 8-hydroxy-2’-deoxyguanosine were found to be significantly elevated in diabetic patients as compared with nondiabetic control and a significant correlation was recorded between them (r = 0.6, p < 0.0001) (Mukhtar et al., 2016). Notably, 8-iso-PGF2α was also associated with patients’ age (r = 0.394, p < 0.0001).

### Metabolic syndrome

In 2010, Renke et al. proved in a randomized, placebo controlled, cross-over study that patients presenting with metabolic syndrome in the course of chronic kidney disease, excreted significantly less isoprostanes with urine after a prolonged 8-week treatment with 40 mg atorvastatin as combined with standard angiotensin converting enzyme inhibitors (ACEI) and/or angiotensin receptor blockers (ARB) (Renke et al., 2010b). Importantly, there were no changes observed in systemic blood pressure, eGFR and serum creatinine. It strongly suggests that normalization of the liver function in terms of cholesterol and triacylglycerols metabolism with a consequent improvement of the plasma lipoprotein profile, contributed to a significant decrease in the overall oxidative stress level in the body. It is obviously believed beneficial for the patients reducing their risk of further metabolic deterioration potentially leading to serious cardiovascular complications, diabetes mellitus, impaired kidney function, or in the long run even cancer development. Another clinical study showed positive association of urinary isoprostane levels with BMI, waist circumference, diastolic blood pressure, and current smoking all of which are well known risk factors of metabolic syndrome and consequent cardiovascular disorder (Anderson et al., 2018b). Urinary F2-isoprostanes were also measured in urine samples collected from 912 premenopausal women aged 35-54 years. Physical activity, alcohol consumption and dietary intakes were self-reported via questionnaires. Significant inverse associations were found between F2-isoprostane and physical activity, vegetables and fruits intake, supplementation with vitamin C, vitamin E, beta-carotene, vitamin A, Se, lutein plus zeaxanthin as well as long-chain omega-3 (ω-3) fatty acids. Interestingly, *trans* fats were positively correlated with F2-isoprostane levels (Anderson et al., 2016). These findings strongly suggest that lower intake of antioxidant nutrients and higher intake of *trans* fats may be associated with greater oxidative stress among premenopausal women increasing the risk of cardiovascular pathology. In addition to that, Zhao et al. found an inverse relationship of urinary F2-IsoP and its peroxisomal beta-oxidation metabolite, 2,3-dinor-5,6-dihydro-15-F2t-isoprostane (F2-IsoP-M) relative to percentage of weight loss as observed in middle-aged and older women (Zhao et al., 2024).

### Cardiovascular disease

There are many clinical reports pertaining to ROS participation in the pathomechanism of cardiovascular disorders, and specifically affecting the condition of the heart muscle. In an article published in Hypertension in 2008, Renke et al. referred to the notion of treatment with a powerful aldosterone antagonist, spironolactone, correlating with a significant reduction in the progression of cardiac hypertrophy and oxidative stress in the heart as observed in uremic rats. As a matter of fact, they managed to prove in a clinical trial that administration of 25 mg spironolactone to patients with nondiabetic proteinuric chronic kidney disease together with standard nephroprotective therapy, significantly reduced urinary levels of 8-iso-PGF2α isoprostane as compared to the control group (Renke et al., 2008). This finding is of clinical relevance, because 8-iso-PGF2α is a biologically active potent renal vasoconstrictor causing arterial hypertension and deteriorating kidney performance. In addition to that, it is expected that lowering ROS generation in the heart muscle by spironolactone administration will have a beneficial cardioprotective effect, and specifically in patients with chronic kidney disease. Interestingly, there are actually quite a few antihypertensive drugs acting on the rennin-angiotensin-aldosterone system (RAAS) which have been proven in clinical trials to effectively lower oxidative stress level as measured by urinary isoprostane excretion (Renke et al., 2009; Renke et al., 2010a).

### Chronic kidney failure

There are many therapeutic strategies adopted for treatment of chronic kidney failure. Importantly, it should be taken into account that patients presenting with an advanced decrease in glomerular filtration rate are very sensitive to a potential increase in ROS generation in the kidneys. Not only can local oxidative stress enhance progression of the disease, but certain products of lipid peroxidation, referred to as isoprostanes being well-known vasoconstrictors, can directly and instantaneously decrease the filtration rate, and thus worsen the overall clinical condition of a renal patient. As certain drugs administered either alone or interacting with others in combined therapies, were proved to increase oxidative stress, it is worth considering to select such nephroprotective medicines which do not induce OS or even better, which act as antioxidants (Renke et al., 2009; Renke et al., 2008; Renke et al., 2010a; Renke et al., 2010b). Aliskiren was introduced to the market in 2011 as the first orally bioavailable direct renin inhibitor approved for the treatment of hypertension. In 2014, a randomized, double-blind trial was performed in 14 patients receiving 300 mg aliskiren with the end point defined as a change in the urinary excretion of N-acetyl-β-D-glucosaminidase (NAG) and α1-microglobulin (α1m) as markers of early tubular dysfunction, as well as 8-iso-PGF2α isoprostane as the biomarker of oxidative stress (Renke et al., 2014). Aliskiren reduced excretion of 8-iso-PGF2α and α1m as compared to placebo. Not only attenuated it oxidative stress but also convincingly improved the functional status of renal tubules in patients with nondiabetic chronic kidney disease (NDCKD). An important practical conclusion from the study is that isoprostanes may be used for diagnosis and monitoring of a disease but also as a clinical tool allowing to choose the optimal therapeutic strategy avoiding OS-related adverse effects or even enhancing the beneficial outcome by decreasing ROS generation.

## Isoprostanes in pregnancy

### Preeclampsia

Isoprostanes are reliable biomarkers of mild oxidative stress that rise naturally during pregnancy due to increased metabolic demand, peaking near delivery. Elevated levels beyond normal physiological ranges are strongly linked to high-risk complications, including preeclampsia, intrauterine growth restriction (IUGR), and fetal growth restriction (FGR), often appearing early as indicators of increased placental oxidative stress (Palm et al., 2009; Palm et al., 2013).

A rise in arterial blood pressure often combined with proteinuria and edema observed in the course of pregnancy has been clinically defined as preeclampsia which if not treated may further develop into eclamptic seizures known as eclampsia, being a direct threat to the life of the mother and the child. Preeclampsia is diagnosed in 3%–8% of pregnancies which is pretty disturbing taking into account severe consequences of this clinical condition. There are many theories of preeclampsia pathogenesis, however, they usually have one mechanism in common which is actually increased oxidative stress involving pathologically elevated lipid peroxidation in the placenta. In 2015 Zabul et al. conducted a study on a group of northern European population of pregnant Caucasian women suffering from limited sun exposure during the winter season (Zabul et al., 2015). They proved that those who developed severe preeclampsia to such an extent that the pregnancy had to be terminated by C-section, excreted substantially higher amounts of isoprostanes in the urine as compared to the control group also undergoing C-section, however, for a different reason with neither arterial hypertension nor detectable proteinuria. Molecular analysis of the potential protective effect of vitamin D3 against oxidative stress phenomena in the placental cells, suggests that high-dose supplementation with vitamin D3 during pregnancy as recommended these days by more and more practicing obstetricians and gynecologists based on an increasing body of biomedical evidence, seems fully justified. The authors proposed that vitamin D3 acts as a competitive inhibitor of the placental cytochrome P450scc, importantly stabilizing this heme-containing enzyme and preventing the production of potentially destructive lipid peroxides and low-molecular electrophiles as well as excess progesterone synthesis, which may all contribute to the etiopathogenesis of preeclampsia.

Similar observations were made in another study comprising sixty pregnant women, including thirty diagnosed with preeclampsia and thirty who were healthy. It turned out that expression levels of serum IsoPs in the preeclamptic group were significantly higher than in the control. Interestingly, the plasma IsoP levels tended to drop by twofold right after delivery (Boldeanu et al., 2023). A most recent clinical trial performed by Dijmarescu et al. seems to confirm that isoprostane (8-epi-PGF2 alpha) level measured by Enzyme-Linked Immunosorbent Assay (ELISA) method in plasma can be utilized as an independent biochemical factor to help clinicians identify or predict which pregnant women will develop preeclampsia (Dijmarescu et al., 2024).

## Isoprostanes in inflammatory and respiratory diseases

### Asthma, sarcoidosis and chronic inflammatory diseases

A study on isoprostanes in childhood asthma was carried out in 54 children, including 12 healthy children, 12 steroid-treated asthmatic children and 30 children with stable asthma (Baraldi et al., 2003). Concentration of 8-IsoP was measured in exhaled breath condensates. IsoP was detected in each group but was significantly higher in both groups of asthmatic children as compared with the healthy children (Baraldi et al., 2003). Psathakis et al. measured 8-isoprostane in the expired breath condensate, as a totally noninvasive method, in order to explore the level of inflammation in pulmonary sarcoidosis. The obtained data showed that 8-isoprostane levels were increased in the expired breath of patients with sarcoidosis and might serve as an index of disease activity (Psathakis et al., 2004). Experimental and clinical studies have revealed that both F2-isoprostanes are involved in severe acute or chronic inflammatory conditions such as rheumatic diseases, asthma, risk factors of septic shock and many others (Basu, 2010; Kadiiska et al., 2005b). In order to examine the role of cyclooxygenase (COX) isozymes in prostaglandin formation and oxidative stress in inflammation, McAdam et al. administered to volunteer subjects placebo or bolus injections of lipopolysaccharide (LPS), which caused a dose-dependent increase in temperature, heart rate, and plasma cortisol. LPS increased urinary prostaglandin levels of iPF(2alpha)-III, iPF(2alpha)-VI, and 8,12-iso-iPF(2alpha)-VI, as well as isoprostane (IsoP) as indices of nonenzymatic lipid peroxidation (McAdam et al., 2000).

## Isoprostanes in cancer and aging

Extensive research during the past decades has revealed that continued oxidative stress can lead to chronic inflammation, which in turn could mediate most chronic diseases including cancer (Reuter et al., 2010). It is believed that high generation of reactive oxygen species in the human body can activate multiple transcription factors such as NF-κB, AP-1, p53, HIF-1α, PPAR-γ, and Nrf2 leading consequently to the expression of over 500 different genes, including those for growth factors, inflammatory cytokines, chemokines, cell cycle regulatory molecules. Overall, observations to date suggest that oxidative stress, chronic inflammation, and cancer are closely linked (Reuter et al., 2010; Nichols et al., 2017).

### Lung cancer

In 2022, researchers from China and USA, published an article with the case-control study of lung cancer, where the aim was to develop a method for measuring 8-IsoP-F2α, as an established marker of lung cancer progression (Ma et al., 2022). They compared the levels of IsoP between three groups: 49 lung cancer patients, 55 benign lung nodule patients and 41 patients with chronic obstructive pulmonary disease (COPD) or asthma. Levels of 8-IsoP-F2α were measured in the blood using ultra-high-performance liquid chromatography coupled with triple quadrupole mass spectrometry (UPLC-tandem mass spectrometric) method. They found significant differences among these groups and suggested 8-IsoP as a marker for early lung cancer screening (Ma et al., 2022). More than 8,000 older adults from the German ESTHER cohort were followed up for cancer incidence by cancer registry data. During 14-year follow-up, 207 lung cancer cases were detected. 8-Isoprostane concentrations were positively associated with the lung cancer (Gao et al., 2018).

### Colorectal cancer

Rasool et al. published an article on IsoPs as biomarkers of OS playing a potential role in the development of colorectal cancer (CRC) in males (Rasool et al., 2018). The levels of isoprostanes in blood samples from 50 CRC patients and 20 control patients were compared. The result showed that the level of IsoPs increased due to high lipid peroxidation in the group of cancer patients. The study also suggested isoprostanes as a prognostic biomarker of CRC progression (Rasool et al., 2018). However, the German ESTHER cohort study following up for cancer incidence did not confirm positive correlation of IsoPs with the incidence of colorectal cancer (Gao et al., 2018).

### Breast cancer

In 2017, Nichols et al. described a correlation between OS and breast cancer in premenopausal women (Nichols et al., 2017). They analyzed 457 cases and 910 controls measuring urinary levels of F2-IsoP and its metabolites. They did not observe a strong correlation between OS as measured by F2-IsoP and the breast cancer risk during menopause. Surprisingly enough, they reported that a higher OS level was associated with an estimated 24%-42% lower breast cancer incidence risk in a group of lean women before the menopausal phase (Nichols et al., 2017). They hypothesized that mildly higher oxidative stress level and the effect of gradual senescence, may sometimes have a beneficial effect on cancer prevention via induction of apoptosis leading to autoelimination of cancerously transforming breast ductal epithelial cells. Over the past several years, a number of studies have consistently observed that overweight or obese women had a significantly elevated level of F2-IsoPs, suggesting that women with a high level of BMI have an excessive production of ROS and are at high risk of clinical complications due to chronic oxidative stress. Indeed, among both overweight and obese women, high levels of F2-IsoPs seemed be related to an increased risk of breast cancer (Dai and Zhu, 2009).

### Prostate cancer

Brys et al. published an article focusing on the relationship of urinary isoprostanes and prostate cancer occurrence (Brys et al., 2013). They estimated oxidative stress in patients with prostate cancer and in the control group. They used 8-iso-PGF2 as a biomarker of lipid peroxidation. They collected urine samples of 304 patients with prostate tumors and 233 urine samples from healthy individuals as control. They observed statistically significantly increased level of IsoPs in the urine from patients with prostate cancer as compared with the control group. These observations were not really confirmed by Gao et al., who discovered a protective association of increasing 8-isoprostane levels relative to prostate cancer incidence, however, this association was only statistically significant among current smokers (Gao et al., 2018). Surprisingly, high oxidative stress level may be a protective factor for prostate cancer.

### Oxidative stress and aging

OS is typically associated with aging (Mukhtar et al., 2016; Liguori et al., 2018; Liu et al., 2023; Chen X. et al., 2021). There are many reasons and hypotheses aiming at the explanation of such a correlation. One of them is the mitochondrial theory of aging which claims that with time passing an inevitable accumulative oxidative damage to the mitochondrial DNA results in multiple mutations within the mitochondrial DNA pool, ultimately increasing the leakage of free electrons from the electron transport chain, and consequently producing much higher levels of superoxide anion in the cell with the following ROS generation cascade (Amorim et al., 2022; Loeb et al., 2005). And thus, OS gradually increasing during aging naturally affects different organs and tissues, however, it is relatively easy to observe the biological consequence of the phenomenon on the surface of the skin. Lipid peroxidation going on within the lipid-rich layers of the skin and subcutaneous tissue, is one of the critical mechanisms responsible for the propagation of ROS generation further degrading the extracellular matrix components, especially collagen and elastic fibers, causing wrinkle formation (Rinnerthaler et al., 2015; Briganti and Picardo, 2003). Analogously, OS-related change in the keratinized stratified squamous epithelium, often becomes the origin of photocarcinogenesis resulting in various types of benign and malignant epidermal tumors (Niki, 2015; Belli et al., 2005) which again, for obvious reasons are not that hard to spot, however, still often are neglected or misdiagnosed worsening a patient’s overall prognosis (Rinnerthaler et al., 2015).

## Isoprostane measurement significance in transplantology

De Las Heras-Gómez et al. conducted a study assessing levels of some IsoPs in the evaluation of the allograft function in patients after renal transplantation (De Las Heras-Gomez et al., 2017). They measured the levels of lipid peroxidation products like F4-neuroprostanes, F3-neuproprostanes (n-6 DPA), F2-dihomo-isoprostanes, F2-isoprostanes and F2-isoprostane isomers. They analyzed the urine of 60 renal recipients by ultra-high-performance liquid chromatography coupled with triple quadrupole mass spectrometry (UHPLC-QqQ-MS/MS). The analysis was conducted at 6 different times during the first 6 months after renal transplantation and the subjects were compared with the control group of 60 healthy patients. A noticeable decrease was observed in the post-transplantation group specifically in three metabolites: 4-epi-40F 3t-NeuroP (n-6 DPA), ent-7 (RS)-7-F2t-dihomo-IsoP and ent-7(S)-7-F2t-dihomo-IsoP. These results suggest that those biomarkers can be useful to estimate renal function during the post-transplant period. Lauzurica et al. documented higher plasma levels of different IsoPs isomers in 43 hemodialyzed patients undergoing kidney transplantation as compared to 50 healthy people in the control group (Lauzurica et al., 2005). Within the three following months, IsoPs levels significantly lowered in transplant patients as compared to the values measured prior to the transplantation procedure. However, in a short study on kidney recipients, urinary IsoPs measured in 11 patients only for 5 days after the transplantation procedure did not statistically differ from the values taken in 11 healthy volunteers (Cracowski et al., 2001).

## Isoprostanes as biomarkers of oxidative stress in skin disorders

Skin is an important physiological barrier, providing a protective envelope between the surrounding environment and the organism. However, human skin is a major target for toxic substances in the form of a broad spectrum of chemicals as well as physical factors (for example, UV radiation) which can change the structure and function of the skin. Some environmental pollutants are oxidants and can directly or indirectly catalyze production of ROS. It is believed that ROS can further activate uncontrolled cellular proliferation caused by UVA-induced mutations leading to the pathogenesis of skin diseases such as photosensitivity disorders (Chen X. et al., 2021; Chen J. et al., 2021) as well as some cutaneous malignancies (Belli et al., 2005). ROS play important roles in pathologic processes including atherosclerosis, ischemia injury and inflammatory response. The skin is able to interact with toxicants by applying defense mechanisms, like enzymatic and nonenzymatic based on low-molecular compounds with antioxidative potential (Briganti and Picardo, 2003). These protective mechanisms, however, have limited capacity which at some point may result in an increased ROS generation promoting the development of skin disorders like: psoriasis, atopic dermatitis, alopecia, dandruff and seborrheic dermatitis (Liu et al., 2023; Chen X. et al., 2021; Belli et al., 2005; Chen J. et al., 2021; Trueb, 2021). Dietary antioxidants and naturally derived bioactive compounds have also been proposed as supportive factors potentially contributing to skin protection against oxidative damage, inflammation and impaired repair processes (Tabrizi et al., 2019; Pasupuleti et al., 2017; Michalak, 2022).

### IsoPs in nonmelanoma skin cancer

Interestingly, nonmelanoma skin cancer is the most common cancer worldwide (Freitas et al., 2015). Total plasma antioxidant capacity was measured in the nonmelanoma skin cancer patients and simultaneously its relationship with food consumption of antioxidant nutrients was analyzed (Freitas et al., 2015). The authors conducted their study including 84 patients: 60 patients after surgery due to nonmelanoma skin cancer and 24 controls. One of the compounds measured in the blood was F2-IsoP. Cancer patients had significantly higher plasma levels of F2-IsoP with no statistically significant difference in the total plasma antioxidant potential as compared to the control group. The research team also showed a correlation between markers of OS and certain antioxidant nutrients in the diet and claimed that food intake of vitamins A and E had reduced OS by removing the negative impact on the redox balance of the skin. As a matter of fact, there are only very scarce publications mentioning IsoPs evaluation in context of the skin cancer patients. Belli et al. identified a higher content of IsoPs in the skin biopsy samples from patients with basal cell carcinoma as well as in the UV-irradiated skin (Belli et al., 2005). In 2015 Freitas et al. proved high 15-F2t-isoprostane plasma levels in patients with a previous history of nonmelanoma skin cancer, suggesting that oxidative stress might play an important role in the pathogenesis of skin malignancies (Freitas Bde et al., 2015). The researchers tried to lower the level of OS in the study cohort by supplementation with both vitamin C and vitamin E, to no avail. There are, however, studies proving such approach successful with four-fold higher vitamin C dose of 2 g per day (Reilly et al., 1996).

### Skin cancer in transplant recipients

It is well known that organ recipients tend to develop different types of cancer at a higher incidence as compared to control population which seems to be directly associated with post-transplant immunosuppression. Skin cancers–squamous cell carcinoma (SCC) and basal cell carcinoma (BCC) – are the most common cancers in kidney transplant recipients and account for over 90% of all skin cancers occurring in this population (Imko-Walczuk et al., 2012; Euvrard et al., 2003). There is no information in the available biomedical literature, however, about potential correlation of IsoPs with skin cancer after organ transplantation.

## IsoP measurement techniques. Availability and applicability consensus

In 2018 European Food Safety Authority (EFSA) claimed that F2-IsoP was the gold standard among lipid peroxidation marker (Turck et al., 2018). They recommended using chromatographic techniques (gas or liquid) with mass spectrometry preferably in 24-h urine collection, which seems to be a better matrix than plasma. Urinary F2-isoprostanes are generally considered more reliable biomarkers of systemic oxidative stress than plasma measurements because they reflect integrated whole-body lipid peroxidation over time and are less susceptible to *ex vivo* oxidation artifacts occurring during blood collection and processing (Turck et al., 2018). In 2022 Król et al. published a review article about methods of identifying IsoPs and utilizing them in clinical medicine as biomarkers. Among 63 publications only 10 of them were based on the recommended chromatographic techniques (Zhao et al., 2024; Krol and Brzeznicki, 2023). LC-MS/MS, as a method for determining IsoPs, allows for simultaneous identification of oxidative stress biomarkers as well as different chemical factors involved (Belli et al., 2005; Turck et al., 2018; Krol and Brzeznicki, 2023). Immunochemical or radioimmunochemical methods for IsoPs evaluation in biological fluids and tissue samples are still widely used in diagnosis and monitoring of OS-related pathologies, however, sometimes combined with more specific gas chromatography and mass spectroscopy methods for validation (Boldeanu et al., 2023; Dijmarescu et al., 2024; Belli et al., 2005; Freitas Bde et al., 2015). Such assays are very straightforward and relatively cheap, as they do not demand sophisticated and costly apparatus, and very importantly, they have been well standardized over the years and are commonly accepted as reliable enough in the clinical context. Table 1 summarizes representative clinical studies evaluating IsoPs as biomarkers of oxidative stress across different pathological conditions.

**TABLE 1:** Representative clinical studies evaluating isoprostanes in oxidative stress-related diseases.

Disease/Condition	IsoP biomarker	Biological material	Main observation	References
Type 1 diabetes mellitus	8-Isoprostane	Blood/EBC	Elevated blood IsoP levels; EBC less suitable for monitoring oxidative stress	Pękala-Wojciechowska A et al. (2018)
Type 2 diabetes mellitus	8-Isoprostane	Plasma	Elevated IsoPs	Mukhtar et al. (2016)
Metabolic syndrome	F2-IsoPs	Urine	Decreased urinary IsoPs after atorvastatin therapy, Increased IsoPs with high BMI and blood pressure	Renke et al. (2010b) Anderson et al. (2018b)
Chronic kidney disease/cardiovascular disease	8-iso-PGF2α	Urine	Reduced oxidative stress after spironolactone and RAAS-targeted therapy	Renke et al. (2009); Renke et al. (2008); Renke et al. (2014); Renke et al. (2010a)
Preeclampsia	8-epi-PGF2α	Plasma/urine	Elevated IsoP levels associated with disease severity and placental oxidative stress	Zabul et al. (2015) Boldeanu et al. (2023) Dijmarescu et al. (2024)
Asthma	8-Isoprostane	Exhaled breath condensate	Higher IsoP levels in asthmatic children	Baraldi et al. (2003)
Pulmonary sarcoidosis	8-Isoprostane	Expired breath condensate	Elevated oxidative stress marker levels	Psathakis et al. (2004)
Lung cancer	8-IsoP-F2α	Plasma/Urine	Elevated. Potential biomarker for early lung cancer screening.	Ma et al. (2022) Gao et al. (2018)
Colorectal cancer	Isoprostanes	Blood/Urine	Increased blood lipid peroxidation markers in colorectal cancer patients. Nonsignificant urinary levels.	Rasool et al. (2018) Gao et al. (2018)
Breast cancer	F2-IsoPs	Urine	Complex relationship between oxidative stress, BMI and breast cancer risk	Nichols et al. (2017) Dai and Zhu (2009)
Prostate cancer	8-iso-PGF2α	Urine	Elevated urinary IsoPs in prostate cancer patients	Brys et al. (2013)
Nonmelanoma skin cancer	15-F2t-IsoP/F2-IsoPs	Plasma/skin biopsy	Elevated oxidative stress markers in patients with skin cancer	Belli et al. (2005) Freitas et al. (2015); Freitas Bde et al. (2015)
Kidney transplantation	F2-IsoPs	Plasma/urine	Oxidative stress decreases after renal transplantation	De Las Heras-Gomez et al. (2017) Lauzurica et al. (2005)

Taken together, the studies discussed in this review suggest that elevated IsoP levels may reflect a common pathophysiological background involving oxidative stress, chronic inflammation, membrane lipid peroxidation and disturbed redox homeostasis. Although the clinical manifestations differ among metabolic, cardiovascular, inflammatory and neoplastic diseases, increased ROS generation appears to be a shared mechanism contributing to IsoP formation. This may explain why IsoPs are repeatedly identified as useful biomarkers across multiple disease entities.

## Conclusion

Oxidation of polyunsaturated fatty acids (PUFA) may change chemical structure and biological activity of phospholipids. Literature reports have indicated that IsoPs being products of PUFAs’ peroxidation can be used as biomarkers in many diseases. In the last 30 years, a lot of studies established possible pathways for IsoPs synthesis, as well as various measurement methods discussing their availability and applicability in the evaluation of OS level.

The review emphasizes representative studies of particular clinical relevance, supplemented by the authors’ own research experience in IsoP assessment in biological material during various clinical investigations. Special focus was placed on the still limited literature data concerning IsoPs as oxidative stress biomarkers in skin cancer, particularly among organ transplant recipients exposed to long-term immunosuppression. Further clinical and mechanistic studies are required to better define diagnostic, prognostic and therapeutic relevance of isoprostanes, particularly in transplant patients who are at elevated risk of skin malignancies.
